# Microplastics in the Environment: Intake through the Food Web, Human Exposure and Toxicological Effects

**DOI:** 10.3390/toxics9090224

**Published:** 2021-09-16

**Authors:** Concetta Pironti, Maria Ricciardi, Oriana Motta, Ylenia Miele, Antonio Proto, Luigi Montano

**Affiliations:** 1 Department of Medicine Surgery and Dentistry “Scuola Medica Salernitana”, University of Salerno, Via S. Allende, 84081 Baronissi, Italy; cpironti@unisa.it (C.P.); mricciardi@unisa.it (M.R.); 2 Department of Chemistry and Biology, University of Salerno, Via Giovanni Paolo II, 84084 Fisciano, Italy; ymiele@unisa.it (Y.M.); aproto@unisa.it (A.P.); 3 Andrology Unit and Service of Lifestyle Medicine in UroAndrology, Local Health Authority (ASL) Salerno, Coordination Unit of the Network for Environmental and Reproductive Health (Eco-FoodFertility Project), “S. Francesco di Assisi Hospital”, 84020 Oliveto Citra, Italy; 4 PhD Program in Evolutionary Biology and Ecology, University of Rome “Tor Vergata”, 00133 Rome, Italy

**Keywords:** microplastic, nanoplastic, environment, food web, human exposure, toxicological effects

## Abstract

Recently, studies on microplastics (MPs) have increased rapidly due to the growing awareness of the potential health risks related to their occurrence. The first part of this review is devoted to MP occurrence, distribution, and quantification. MPs can be transferred from the environment to humans mainly through inhalation, secondly from ingestion, and, to a lesser extent, through dermal contact. As regards food web contamination, we discuss the microplastic presence not only in the most investigated sources, such as seafood, drinking water, and salts, but also in other foods such as honey, sugar, milk, fruit, and meat (chickens, cows, and pigs). All literature data suggest not-negligible human exposure to MPs through the above-mentioned routes. Consequently, several research efforts have been devoted to assessing potential human health risks. Initially, toxicological studies were conducted with aquatic organisms and then with experimental mammal animal models and human cell cultures. In the latter case, toxicological effects were observed at high concentrations of MPs (polystyrene is the most common MP benchmark) for a short time. Further studies must be performed to assess the real consequences of MP contamination at low concentrations and prolonged exposure.

## 1. Introduction

Plastics are widely employed in many applications, ranging from food packaging to technological devices and disposable medical equipment, thus making them present in everyday human life. However, the consequential human exposure to microparticles derived from plastic materials could have, over time, harmful effects. In literature, a large number of studies are dedicated to the transport of microplastics in the food web through air, water, and soil environments; their persistent nature can be very toxic to humans. Plastic debris is defined as microplastics (MPs) by the National Oceanic and Atmospheric Administration (NOAA) when the particles have a diameter lower than 5 mm. The classification of microplastics is also based on their source: microplastics are defined as primary if released intentionally in the environment and secondary if they are released indirectly by deterioration processes. Microbeads and abrasives in personal care products and cleaning formulations are examples of primary MPs intentionally included in products and used in the manufacturing of plastic materials [1]. Secondary microplastics can derive from the deterioration, fragmentation, or improper disposal in the environment of large pieces of plastic, such as plastic films, household garbage, atmospheric deposition, and vehicle emissions [2,3,4]. Mixtures such as paints can release both primary and secondary microplastics: primary when the paint is in its fluid form and secondary if small particles detach from the dried paint (for example, fragments of ships and boats) [5].

Microplastics can have different shapes (fibers, fragments, spheres, beads, films, flakes, pellets, and foam) depending on the original form of the plastics, the deterioration processes occurring on the plastic surface, and the residence time in the environment [6,7,8]. The potential of microplastics to cause physical harm to organisms is affected by their size and shape [9]. In fact, although large microplastics are not taken up by most plants and soil organisms, small particles (e.g., nanoplastics) can be easily taken into cells, thus generating an environmental risk [10,11].

Concerning plastic shape, some studies suggest that fibers are more toxic on marine invertebrates with respect to fragments and spheres having the same polymer matrix [12,13]. In addition to petroleum-based plastic fibers, man-made cellulose fibers (e.g., viscose/rayon) have also been detected in different environmental matrices (deep-sea sediment [14], macroinvertebrates [15], fishes [13,16]), thus increasing the interest of the scientific community in this kind of plastic pollution, which is usually underestimated. This type of fiber is biodegradable in the natural aquatic environment, so it is not considered an environmental issue in itself. However, the additives it contains may be harmful to aquatic organisms.

In fact, the presence of organic and inorganic additives and traces of monomers, metals, or other compounds that can be released represents a more toxic source of pollution for human health than the MP fragments themselves [17,18,19]. For example, chemicals such as bisphenol A [20] and phthalates [21] are often found in association with microplastics; these endocrine disruptors can be very hazardous for humans [22,23,24]. Other adverse effects on the environment derive from the fact that the MPs can also act as vectors for other contaminants [25] (e.g., potential human pathogens [26,27,28], organic pollutants [29], heavy metals [30,31,32,33]). In fact, the adsorption of persistent organic pollutants (POPs), mainly polycyclic aromatic hydrocarbons (PAHs), polychlorinated biphenyls (PCBs), polybrominated diphenyl ethers (PBDEs), and dichlorodiphenyltrichloroethane (DDT) on microplastics has been reported [29]. Trace elements were found in combination with microplastics in the marine zooplankton of the Mediterranean Sea. Aluminum, iron, chromium, zinc, nickel, molybdenum, manganese, lead cobalt, and copper were found at concentrations of mg/kg while arsenic, vanadium, rubidium, and cadmium at level of μg kg^−1^ [30]. The levels of aluminum, copper, and zinc registered were comparable with the values found in microplastics collected in England and Brazil, while the levels of iron and manganese were lower in the samples collected in the Mediterranean Sea [31,32,33].

Microplastics may concentrate in the human body through various exposure pathways (see paragraph on “Implication of microplastic contamination on human health”), such as inhalation of dust and direct consumption of food contaminated by microplastics. In fact, the maximum estimated intakes of microplastic from dust ingestion for adults and children are about 1000 and 3000 particles per year, respectively [34]. At present, knowledge of the effects and toxicity of microplastics to humans is very limited, and the research on the trophic transfer of microplastics in the food web to humans is key to preventing microplastic contamination problems.

This review covers some environmental routes (water, air, and soil) of microplastics contamination into the food web, describing their effects on human health, and presents new and relevant studies on their occurrence, fate, and behavior.

## 2. Methodology

The authors thoroughly reviewed the literature related to microplastics, finding that current research is predominantly focused on environmental contamination rather than human health interactions. This review went through under a three-pillar approach: (1) delineation of the urgency and seriousness of microplastics in the food web by emphasizing the various ways that microplastics interact with the human body; (2) impacts of microplastics on water, air, and soil properties through multiple aspects, as well as the potential risks when dispersed into other environment media, transferred along the food chain, and accumulated by animals, plants, and humans; (3) determination of contamination and accumulation of MPs in water, soil, air, and food, particle toxicity, and the proposal of future research directions according to the existing literature. The keywords “microplastic”, “environment”, “food web”, “human exposure”, and “toxicological effects” were selected individually or jointly to search for relevant information on Web of Science, Scopus, and Google Scholar. Key literature published between 2004 and 2021 (up to June) were assimilated and analyzed.

## 3. Occurrence, Analysis, and Abundance of Microplastics in the Environment

Microplastic pollution was first observed in the marine environment: in the 1970s, spherules, disks, and pellets were detected on the surface of the Sargasso Sea [35], on the coasts of New England [36], in the surface waters of the Atlantic Ocean [37], and in the surface waters of the Pacific Ocean [38]. Recently, the attention of researchers has moved to wastewater, rivers, and lakes [39,40,41,42,43]. Due to the increasing interest in this field, several review articles [3,44,45,46,47,48,49] were published in the last few years, with the aim of giving an overview of microplastic presence in different water matrices all over the world, analytical methods for their detection, and possible consequences to human health [2,7,39,50,51]. The occurrence of microplastics in the water environment was widely discussed in our recently published review, which focused on their abundance in different water matrices and their distribution worldwide [52]. In contrast, the present review mainly deals with the transport of this contaminant to humans through the food web, with only an introduction of the pollution of the different environmental compartments.

As regards the origin of MP contamination in water environments, the main sources are improperly disposed plastic wastes from land and, to a lesser extent, derivatives from marine activities, such as the fishing industry employing plastic equipment [51,53]. The fragmentation of plastic debris in water leads to the contamination of aquatic species through active and passive intakes, with consequent transfers within the food web [54].

More recently, microplastics have been recognized as air pollutants [55,56,57,58,59]. Alongside other atmospheric contaminants such as nitrogen oxides [60,61,62], hydrogen sulfide [63,64], carbon dioxide [65,66], persistent organic pollutants [67], and BTEX [68,69], their concentration should be regularly monitored to ensure human safety. Several studies have shown that atmospheric microplastic particles can be carried to remote areas by atmospheric events such as winds [70,71,72,73], ocean currents [2,74,75], river outflow, and drift [76,77]. Airborne MPs may be deposited in aquatic environments and soils via dry or wet deposition [78], with a consequent spatial distribution and temporal variability of their abundance [79,80]. As a consequence, microplastics have been detected even in remote areas such as the Antarctic and the South Indian Ocean.

The estimated amount of annual plastics discharged to the soil is much higher than that released in the oceans [81], so the terrestrial environment can be considered an important sink for microplastics [82,83]. However, microplastic pollution in the soil environment has been largely overlooked for several years [84,85], gaining attention since 2012, when Rilling [86] identified the possibility of microplastic occurrence in the terrestrial environment and the need for studies for their estimation. The low number of investigations is probably due to the unavailability of suitable analytical methods for microplastics in soils [87]. Plastic mulch films, compost and municipal solid waste, biosolids such as anaerobic digestate and sewage sludge, irrigation and flooding of wastewaters, atmospheric deposition, illegal dumping of waste, and plastic-coated fertilizers represent the principal sources of microplastics in soil [81,88,89,90,91,92].

The collection and treatment of samples are the most important steps to obtaining a satisfying determination of microplastic pollution without contamination. Different sampling techniques are required according to the type of environmental matrix considered. For the water compartment, the main sampling methods are sediment recovery from the seafloor, beaches, and estuaries, beachcombing for the shoreline, observation by divers, use of marine trawls to collect particles within the water column, and examination of plastic fragments ingested by marine organisms [2,7].

Regarding atmospheric microplastics, most of the studies have employed a passive sampling of deposited material [93,94,95], while only a few studies have used active sampling [96,97,98], often in comparison with passive ones.

The two sampling methodologies provide different information regarding microplastics in the air. In particular, passive samplers give an estimation of the number of deposited microplastics onto the surface of a certain place during a specific time-lapse, while active devices return the number of microplastics in the air mass that may not be deposited [99].

Once sampling has been carried out, the analysis of MPs can be done through several procedures such as separation, identification, and quantification. Sieves usually achieve the first step of microplastics separation, with mesh sizes ranging from 0.038 to 4.75 mm and filters with small mesh sizes (0.02–5 μm) [100,101]. For small particles (dimensions < 1 μm), many studies have reported the use of active (e.g., field flow fractionation technique) [102] and passive separation (e.g., chromatographic techniques such as hydrodynamic chromatography) [103,104].

Particle visualization, as well as the evaluation of color, shape, and light transmission, is important for identifying microplastics in sample materials or debris (e.g., shell fragments, algae, sand, and glass) [7] and distinguishing plastic from non-plastic particles [105]. Large microplastics (1–5 mm) are identified with the naked eye, and an optical microscope can be used for smaller microplastics to obtain images for analysis, which provide the shape and number of the microplastic particles. More advanced techniques, such as SEM-EDS (scanning electron microscopy coupled with energy-dispersive X-ray spectroscopy) [106], were recently reported in the literature to characterize the morphology of ultra-small plastic particles and facilitate the differentiation of microplastics from other plastic-like particles [107]. Lastly, the characterization of samples in terms of chemical composition, i.e., polymer type, is mainly performed by FT-IR (Fourier transform infrared spectroscopy) and Raman spectroscopy [108]. These are useful techniques for identifying the polymeric composition of microplastics of different types (with sizes ≤ 2 µm) since they require small sample amounts and limited sample preparation. Moreover, these spectroscopies are complementary vibrational techniques. Generally, signals with strong IR intensity have weak Raman intensity and vice versa. For instance, carbonyl groups, being polar functional groups, are well detected by IR, whereas aromatic bonds and double bonds are better identified using Raman [109].

The analytical techniques described in this section are used for the identification of microplastics in all the environmental compartments, i.e., water, air, and soil. The quantification of MPs is still a great challenge due to their special chemical and physical properties (very high molecular weights, poor solubility in most solvents). Although new accurate and sophisticated analytical methods have been reported for microplastic detection and characterization, FT-IR and Raman spectroscopy remain the most commonly used for identifying the polymeric composition of microplastics, accompanied by visual characterization through optical microscopy.

## 4. Microplastic Transport in the Food Web and Consequent Human Exposure

**Figure 1:**
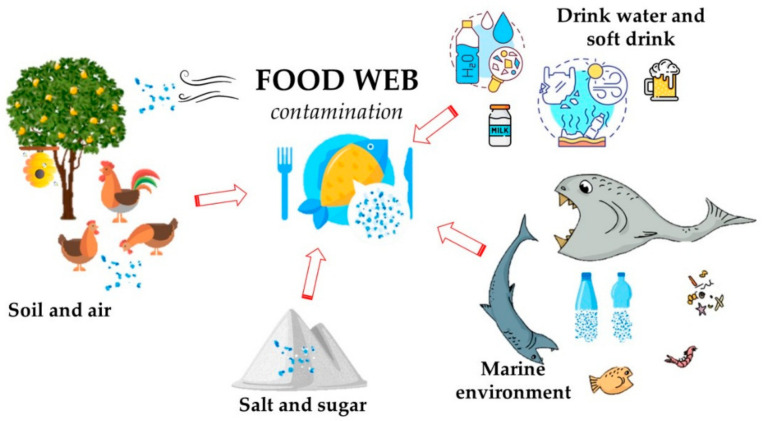
Scheme of food web contamination due to MP pollution.

As reported above, microplastics can be generated through several mechanisms and can be transported across different environmental compartments, reaching the food web and, finally, the human body. Figure 1 shows the main pathways of food contamination through soil/water/atmosphere. Human ingestion of contaminated food and beverages is a concern [110,111], even if it seems to be unappreciated compared to environmental implications.

In this paragraph, we discuss peer-reviewed papers on MP contamination in edible animal species (seafood and chicken) and food samples such as salt, sugar, honey, milk, fruit, soft drinks, and drinking water.

Food and beverage samples must be pretreated to avoid large errors in the results. For example, drinking water samples were filtered, in parallel, through stainless steel filters [112]. A flowmeter was connected to all the outlet tubes of the filtration units to quantify the volume of filtered water. At each sampling position, the setup was primed for ten minutes prior to applying filters. Finally, the filters were transferred to glass Petri dishes, covered with 70% ethanol, and stored at −20 °C until further processing. Salt samples can be treated with hydrogen peroxide to digest possible organic content [113]. Fish samples can be caught using different types of gear and transported to fishing harbors in ice chests and later transferred to another ice chest and stored at −20 °C before the analysis [114]. Similarly, chicken feces and other food samples are frozen before the analysis to avoid decomposition [115].

Sometimes fishes and mussels are collected from the sea and kept alive to study how they interact with MPs. In these cases, they can be held in fiber-glass tanks with artificial seawater to acclimatize to laboratory conditions, and then microalgae or shrimps are added to feed the animals [116,117].

Once the exposure time is completed, it is necessary to extract the MPs and remove fats, proteins, sugars, and other substances through digestion with acids, alkali, oxidizing agents, and enzymes. Sometimes, multiple digestion steps are required to improve the analysis. The analytic techniques for particle characterization and chemical composition are the same discussed in the paragraph “Occurrence, analysis, and abundance of microplastics in the environment”.

### 4.1. Seafood

The marine environment is the most studied system for MP contamination, and sea products (e.g., wild animal species, algae, sea salts) [118] are one of the primary sources of food for humans [119]. Consequently, several studies have investigated microplastic occurrence and abundance in marine species, expressing MP concentration as the number of particles/g or particles/individual [120,121,122,123,124,125,126,127,128,129,130,131,132,133,134,135,136,137,138]. There are two different mechanisms to describe how MPs reach the organs of fish. MPs may be captured actively (by confusion with prey), passively (e.g., gill water filtration), and through the ingestion of contaminated prey [139].

Shellfish (including crustaceans and bivalves) and other fish species are often contaminated with microplastics. Microplastics were detected in the 25 species contributing most to global sea fishing, including the Atlantic cod (*Gadus Morhva*), the European hake (*Merluccius Merluccius*), the red mullet (*Mullus Barbatus*), and the European pilchard (*Sardina Pichardus*) [114]. Miranda and Carvalho-Souza [140] also found microplastics in the digestive tract of two important species of edible fish (*Scomberomorus Cavalla* and *Rhizoprionodon LandII*) from the Brazil’s eastern coast, although, in some cases, this would not be a big health concern as the GI tract is discarded during processing. However, scientific studies have analyzed the accumulation of microplastics in other species such as *D. Labrax*, *T. Trachurus,* and *S. Colias* specimens from Portuguese coastal waters [114,141]. In this case, a different amount of plastic debris that could be explained by various mechanisms of contamination (passive, active, and through contaminated prey) was observed for each species. *S. Colias* showed a higher percentage of microplastic contamination in the gastrointestinal tract (62%) than the other species (42%), probably due to some distinct ecological features (e.g., time spent in areas closer to shore, feeding ecology) and physiological differences (e.g., water filtration rates, elimination processes) [114].

Among the 150 fishes analyzed, the percentage value obtained of 35% is comparable to the corresponding values reported in the literature: 19.8% of 263 fishes from Portuguese coastal waters, 38% of 120 fishes from the Monolego River estuary in Portugal, 58% of 1337 fishes from the Mediterranean Sea, and 65% of 178 fishes from the Red Sea [141]. In the Persian Gulf, a wide variety of pelagic and benthic species was investigated: about 128 marine organisms, divided into 3 fish species, 1 prawn species, and 1 crab species. The results found that the MPs were located in the muscles and gills of the individuals [142]. In this case, the adsorption in muscles could show toxicological effects since all particles were equally transferred to the next level of the food web. Microplastic fibers were recently detected on the external surface and in the gastrointestinal tract of clupeid fishes (larval and juvenile stages) from the Mediterranean Sea, i.e., *Sardina pilchardus* (0.53 items/specimen) and *Engraulis encrasicolus* (0.26 items/specimen). Since these clupeids are among the main food sources for several marine species, these results give rise to concern relating to the possible transfer of microplastics through the marine food web and into humans [109].

The uptake of MPs is also influenced by the chemical and physical composition of water, in particular by salinity. A study of fishes in the Saudi EEZ of the Arabian Gulf, based on 15 individuals of *Lethrinus nebulosus*, 20 *Gerres acinaces*, 20 *Siganus canaliculatus*, 6 *Liza parsia*, 10 *Scomberomorus commerson*, 20 *Euthynnus affinis*, 20 *Epinephelus coioides*, 20 *Rastrelliger kanagurta*, and 9 individuals of *Carangoides malabaricus,* indicated that only 5.71% of samples ingested MPs. The lower value is probably due to the presence of microplastics in the sea surface microlayer and, consequently, less availability for ingestion by fishes [139]. In the marine food web, consumers or predators can ingest MPs through prey items such as polychaetes, mollusks, small crustaceans and arthropods, annelids, and fish larvae. So even though the gastrointestinal tract is discarded during processing, the presence of MPs in the gastrointestinal tract of marine organisms has raised concern worldwide as seafood can be a significant source of MPs in humans [143,144,145,146]. A study on the gastrointestinal tracts of tiger shrimp (*P. monodon*) and brown shrimp (*M. monocerous*), commercially important shellfish species of Bangladesh, evaluated the presence of MPs averaging 3.40 ± 1.23 and 3.87 ± 1.05 particles/g of the gastrointestinal tract; FTIR data confirmed particles of polyamide-6 and rayon polymers, the common raw materials of ropes, fishing nets, floats, fish baskets/bags, and coatings used in the sea [147].

Moreover, the contamination of MPs involved not only fish from the sea; this could be a warning for freshwater fishing and fish farming industries to introduce more controls. Cultured organisms can be exposed to high levels of MPs, as evidenced by the analysis of fish meals from three different Malaysian commercial brands [148]. In the study, a total of 336 particles was isolated, and 64.3% were identified as MPs; micro-Raman spectroscopy confirmed that the most abundant isolated polymer was PE (63.0%), followed by PP (27.8%), PET (8.8%), and NY (0.4%).

A useful tool for quantifying the level of microplastic contamination within the freshwater food web is stable isotope analysis [149]. This technique measures the relative abundance of stable isotopes, giving an isotopic ratio, expressed as δ in ‰ [150,151], and providing information about the origin of a sample [152]. It has been widely used in food analysis [153], medical diagnostics [154], monitoring of air quality [155,156,157], and characterization of commercial cleaning products [158]. It has been recently employed to evaluate the presence of microplastics [159] and to discriminate between polymer sources (petroleum and plant-derived) [160,161]. The carbon stable isotope ratio (δ^13^C) and nitrogen stable isotope ratio (δ^15^N) were used to quantify trophic niches for macroinvertebrates and fish within the Garonne River. The abundance of ingested microplastics varied between macroinvertebrates and fish and was not significantly related to pollution; moreover, it increased with the size of organisms and was affected by the origin of the resources consumed by fish. The authors assert that results of isotopic analysis suggest the absence of microplastic bioaccumulation in freshwater food webs and the dominance of direct (accidental) consumption; therefore, the stable isotopic ratio is very useful for a deeper understanding of microplastic ingestion by wild organisms [149].

These studies will not only be beneficial to understanding the distribution and migration of microplastics in marine ecosystems but also provide an important reference for the protection and governance of seafood.

### 4.2. Salt for Human Consumption

In the last few years, several studies regarding the microplastic contamination of salt intended for human consumption [162,163,164,165,166,167,168,169,170] have been published and discussed [171,172,173,174]. Salts provide essential nutrition elements and, thanks to their chemical characteristics and low cost, are used in food preservation methods (e.g., fruits, cheese, cereals, drinks). Other uses for salt, for example, are in the cosmetic and personal care product industry and the pharmaceutical industry (as an additive, stabilizer, and thickener).

MPs have been found in the commercial salts of 128 brands from 38 different sources in numerous countries spanning over five continents in the period 2015–2018 [173]. MP abundance in table salt was different among the countries under study: the lowest values were detected in China (600 particles/kg) [170] and the United States (800 particles/kg) [166], while higher values were discovered in Italy (8000 particles/kg) [168], Indonesia (10,000 particles/kg) [165], and Croatia (20,000 particles/kg) [168]. The particles identified in sea salt were made of cellulose, cellophane (CPH), polyethylene-vinyl acetate (PEVA), PA, polyacrylonitrile (PAN), polyalkene, poly(1-butene), PET, poly(metylacrylate), PP, phenoxy resin (PR), polyurethane (PU), polyvinyl chloride (PVC), and paraffin wax. The results of a meta-analysis [171] proved that the microplastic content in salt strongly depends on its origin; in particular, sea salt is the most contaminated, followed by lake salt, rock, and well salt. The microplastic contamination of salts can derive from the alteration of larger plastic pieces through biological, photo, and/or mechanical degradation in the environment or from the direct input of particles from industrial processes and the manufacture of a wide diversity of everyday-use products. The high amount of microplastics in sea salt derives from the fact that it is produced from the evaporation of seawater, which often contains harmful microplastics. Hence, recently, a coagulation process was developed for a clean sea salt production by removing microplastics from seawater, showing interesting results [175].

Consequently, maximum human exposure is estimated to be 6110 microplastic particles per year, confirming salt as a microplastic carrier. To illustrate human risk, several authors estimated the annual consumption of microplastic particles through sea salt. The values are 37 [164], 64–302 [162], 40–680 [166], 510 [163], and 1000 particles [170] based on human ingestion of 5 g of salt per day, the recommended intake threshold by the World Health Organization [176]. However, the actual salt intake can be much higher (10 g/day worldwide [177]) than the recommended one, thus increasing the human ingestion of MPs derived from salt.

### 4.3. Drinking Water

Freshwater bodies are the predominant drinking water source for human consumption. In literature, some scientific papers introduced the role of potable water as a suspect potential source of MPs [178,179,180,181,182,183,184,185,186]. The presence of fibers in surface water is, therefore, presumably caused by the inflow of sewage water. Freshwater has been shown to contain PE and PP, comprising up to>90% of MPs in drinking water, and also PET, PS, PVC, polyester (PES), PA, polytetrafluoroethylene (PTFE), and RY, the materials commonly used in various products, in particular, food and cosmetic packaging, houseware, and toys [174]. Some studies analyzed water samples in Germany, including raw water, drinking water, tap water, and bottled water [181,182,184]. Water samples taken at different positions within the drinking water supply chain (raw water and drinking water) have shown an average microplastic concentration of 700 particles/L (range 0–7000 particles/L) [181]. The detected microplastic particles were small fragments (size range 50–150 μm) of polyester, polyvinylchloride, polyethylene, polyamide, and epoxy resin, probably introduced during drinking water purification and transport. Other authors have investigated microplastic abundance in bottled water from diverse packages (single-use plastic bottles, reusable plastic bottles, beverage cartons, and glass bottles); however, no statistical differences were observed among samples [182,184]. Microplastics in the size range of 5–100 μm were detected in concentrations from 11 ± 8 (beverage cartons) to 118 ± 88 (returnable plastic bottles) particles/L [184]. In contrast, a higher microplastic amount in mineral water from reusable PET bottles (average 4889 ± 5432 microplastics/L) compared to single-use PET bottles (2649 ± 2857 particles/L) and also a high microplastic content (3074 ± 2531 microplastics/L) in glass bottles were observed in the size range of 1–5 μm [182]. Microplastic abundance in raw and treated water was determined from three drinking water treatment plants in urban areas of the Czech Republic [183]. Microplastic content was significantly lower in treated (from 338 ± 76 to 628 ± 28 particles/L) compared to raw water (from 1473 ± 34 to 3605 ± 497 particles/L), and most of the particles were within the size range of 1–10 μm.

The detected microplastic concentrations in treated water are not negligible and suggest that potable water could be an important source of microplastics to humans. Recently, the World Health Organization [187] has reported that the microplastic content in tap water is about 5 particles/L, with a consequent daily dose of microplastics of 10 particles, considering a human water intake assumption of 2 L/day [188]. A significant increase in the amount of microplastic intake must be considered in individuals who drink water only from plastic bottles compared to those who consume only tap water (additional 90,000 annual microplastic particles compared to 4000).

### 4.4. Soft Drinks

Beverages intended for human consumption are divided into groups of alcoholic and non-alcoholic drinks. Beers, wines, and spirits are classified as common alcoholic drinks, while non-alcoholic drinks include tea, coffee (hot and cold), soft drinks, milk, chocolate, carbonated, and non-carbonated sweetened drinks.

In Mexico [189], a total of 57 beverage products, including cold tea, soft drinks, energy drinks, and beers, was investigated to develop baseline data on the levels of MPs. Chemical characterization results allow for the distinction of different forms of MPs (fibers and fragments) and various sizes of 0.1–3 mm with different colors (blue, red, brown, black, and green). The chemical nature of MPs obtained by micro-Raman spectroscopy indicated contamination from synthetic textiles and packaging in the beverage products; indeed, the particles identified were PA, poly(ester amide), acrylonitrile, butadiene, styrene, and PET, the most common raw materials of these commercial products [189]. A possible source of contamination could be the water used in the bottling industry production of soft drinks, which comes from various supply sources: groundwater, surface water, a public water network, or rainwater [190]. Considering that the international market of drinking water has an annual volume of over 245 billion liters, small quantities of MPs in drinks can also have harmful effects on human health due to accumulation and interaction with other compounds. An appropriate study in vivo is necessary to preserve and understand all possible implications.

In German beers, a 2014 study investigated microplastic contamination and found fibers, fragments, and granules after filtration through a 0.8 µm cellulose filter, except for wheat beers that could percolate and were filtered through a 40 µm sieve [191,192]. However, there were many criticisms of the results because researchers affirmed that contamination could not have originated from the raw material, but there were artifacts due to laboratory contaminations. In 2018 [166], the MP contamination of American beers was evaluated: the samples were filtered with an 11 µm pore size, and the authors postulate that product processing might be the most important factor explaining human contamination.

### 4.5. Milk

Dairy milk products are a part of globalized commodities for regular income; world exports expanded to 75 million tons (in milk equivalents), and global milk output in 2018 was estimated at 843 million tons [193]. However, the industrial process of milk production has been subjected to many technological developments to improve hygiene and human health, which have influenced milk composition [194]. Considering the intense processing of milk, the possible risks of milk contamination from microplastics may occur from poor cleanliness procedure equipment, the surrounding environment, as well as water supply conditions and the inadequate handling of milk. The presence of MPs in dairy milk products was detected in Mexico [195], where 23 milk samples from 5 international and 3 national brands were analyzed. All samples analyzed included microplastics. Scanning electron microscopy coupled with energy-dispersive X-ray spectroscopy (SEM-EDS) was used to examine the surface morphology and elemental composition of microplastics in milk samples. A total of 150 microplastic particles were confirmed in 23 milk samples, with an average concentration of 6.5 × 10^3^ particles/m^3^, lower than any reported levels in liquid food products, confirming the ubiquity of MPs in samples and showing variability in the range of 3–11 × 10^3^ particles/m^3^ [195]. The most common contaminants in milk samples are the thermoplastic sulfone polymers that are used in the ultrafiltration and microfiltration membranes in food and dairy industry processing. High pressure and continuous chemical and physical stress can damage the membranes, peeling off particles from filters, and, thus, may be a source of microplastics in fluid milk samples. The release of microplastics from milk raises serious concerns because the main consumers of milk are infants.

Infant feeding bottles, commonly made of PP, can release microplastics in milk, as evidenced in the paper of Li et al. [196]. In this study, the authors also estimated the intake of MPs on all the continents. The average daily consumption of PP MPs by infants was estimated to be 1,580,000 particles per capita, with a range of 14,600–4,550,000 particles, depending on the region. Infants in Africa and Asia have the lowest potential exposure to MPs from PP bottles, with rates of 527,000 and 893,000 particles per day, respectively. Infants in South America have medium exposure levels (1,010,000 particles per day), whereas Oceania, North America, and Europe have the highest levels, corresponding to 2,100,000, 2,280,000, and 2,610,000 particles per day, respectively. The average value corresponds to about 3000 times the total adult consumption of MPs from water, food, and air (up to 600 particles per day for adults).

### 4.6. Honey, Sugar, and Fruit

The analysis of 19 samples of honey coming from different countries revealed a high concentration of microplastic particles [197]. In 2019, the Swiss Beekeepers Association started new research to understand the origin of MP contamination in honey. Therefore, they discriminated beehives made of wood from those made of polystyrene to explore the significant contamination source in honey. For these particle classes, they were able to identify the particle materials using attenuated total reflection–Fourier transform infrared spectroscopy (ATR-FTIR) and Raman techniques, which, in some cases, even allowed them to relate the particles to their specific origin. The results do not provide any evidence for notable contamination in honey from environmental sources, but some microscopic particles could be related to beekeeper activities [198]. Microplastics were also discovered in honey from Ecuador, with a concentration of 54 and 67 particles/L in industrial and craft honey, respectively [199]. This microplastic contamination of food products probably originated from atmospheric contribution during production processes.

The mean values of microplastics in sugars (excluding the cane sugar sample) were 217 ± 123 fibers/kg and 32 ± 7 fragments/kg, with the maxima of 388 and 270, respectively. For these samples, no distinction was made between colored and transparent particles. International sugar suppliers usually offer purities ≥ 99.8%, where these sugars will contain a maximum of 0.2% foreign matter. However, despite the large numbers of foreign particles found in both honey and sugars, they will most probably not exceed any governmental or industry limits [197].

Very recently, microplastics were detected, for the first time, in edible fruits (52,600–307,750 particles/g) and vegetables (72,175–130,500 particles/g) in the size range of 1.36–3.19 µm, with apples and carrots being the most contaminated samples (estimated daily intakes of MPs in the range of 2.96 × 10^4^–1.41 × 10^6^ particles/kg/day) [200].

### 4.7. Chicken, Cows, and Pigs

In 2017 [115,201], MP transfer from soil to chickens was investigated for the first time in traditional Mayan home gardens in Southeast Mexico. The authors analyzed the concentration of MPs in soil, earthworm casts, chicken feces, crops, and gizzards (used for human consumption) and observed that the concentrations increased from soil (0.87 ± 1.9 particles/g) to earthworm casts (14.8 ± 28.8 particles/g) to chicken feces (129.8 ± 82.3 particles/g). Chicken gizzards contained 10.2 ± 13.8 microplastic particles/g, while no microplastic was found in crops. Most recently, it has also been reported that microplastic contaminants can be present in chicken meat and feces [202]. Other researchers have evaluated the use of a rapid method based on attenuated total reflection mid-infrared (ATR-MIR) spectroscopy combined with chemometric techniques to identify the level of contamination in chicken meat with microplastics polystyrene (particle size 100 μm) and polyvinyl chloride (particle sizes 3 μm, 100 μm, and 2–4 mm) [203].

The occurrence of microplastics in livestock and manure has been reported in 19 different farms in South China, where pigs, poultry, and cows are raised [204]. PP and PE in colorful fibers and fragment types were the most abundant MPs. Livestock and poultry animals can eliminate MPs when eating polluted feeds. MPs can also reside in the digestive system, despite the high levels of digestive juices; thus, MPs can pass through the digestive system and remain in the manure. After the contaminated feces is used for composting, agricultural soil can be polluted by the MPs present in the compost. Another source of MP contamination for meat is the packaging used: extruded polystyrene microplastics can contaminate meat at 4–18.7 particles per kg of food [205].

These are very small examples of MP contamination in edible animals, and they are not sufficiently representatives of a real meat contamination issue.

### 4.8. Some Considerations of Food Web Contamination

Based on the studies discussed above, great efforts have been devoted to microplastic detection in sea products, drinking water (raw, treated, and stored in different packages), and salts for human consumption, while only a few studies have been conducted on other foods such as honey, sugar, fruit, and chickens. This higher number of studies is probably due to the easy transport of microplastics from a polluted water environment to related food products. Several factors are responsible for an MP presence in food products. The contamination can be related to environmental sources (contamination of water, soils, and air) [197] and manufacturing processes such as the materials used during the filtration step of beer and milk [199].

MP contamination can also be due to packaging, such as bottled drinking water, beer, milk, and refreshments [206], extruded polystyrene for meat [205], and take-out food containers. In a study published in 2020, four plastic containers made of polypropylene (PP), polystyrene (PS), polyethylene terephthalate (PET), and paperboard coated with PE were analyzed: the highest microplastic abundance was found in the containers made of PS [207]. Based on the MP abundance of food containers and take-out order frequency, human microplastic intake is estimated as 12–203 items per week. The packaging material has less effect on beer since, in most cases, beer is packaged in glass bottles and aluminum cans [199].

MP occurrence in food and beverages is recapped in Table 1. The estimated intake of MPs through inhalation and ingestion is reported in Table 2. Among various foods investigated, salt is the one that releases the least microplastics, while a high intake of microplastics can occur through milk stored in PP bottles used for infants. As regards meat and seafood, the dose of MPs ingested depends on dietary habits: for example, in the UK, where people eat less seafood, the MP intake is minor compared to Mediterranean countries [208].

All these studies suggest a not-negligible ingestion of microplastic particles. However, these examples are still too few to be sufficiently representative of true microplastic contamination, so further studies need to be performed in order to have more data on the human ingestion of microplastics from foods different from those related to the water environment. Considering these results, toxicological and epidemiological studies need to be performed to investigate more deeply the possible consequences of microplastics found in foods on human health.

## 5. Implication of Microplastic Contamination on Human Health

### 5.1. Possible Routes for Human Exposure to Microplastics

**Figure 2:**
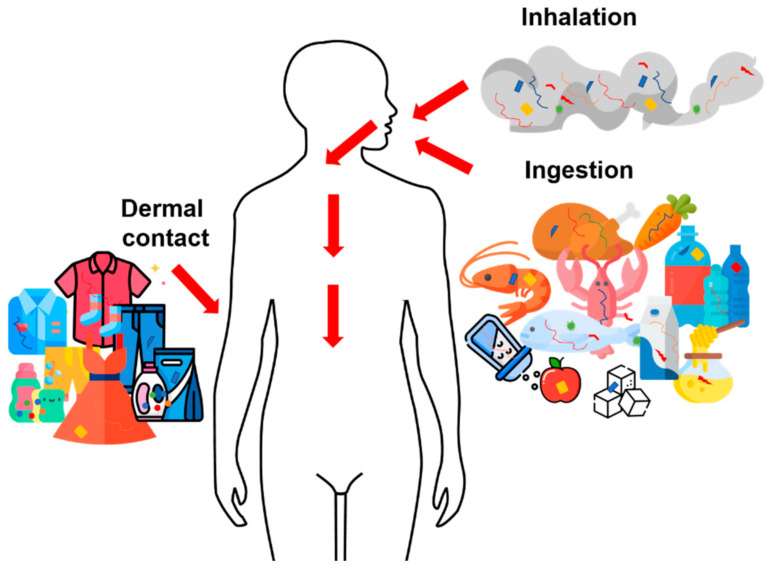
Schematic representation of exposure to microplastics through three routes: ingestion, inhalation, and dermal contact.

The human body’s exposure to microplastics passes through different routes: ingestion [136,188], inhalation [210], and dermal contact [212] (Figure 2). Each of these routes of exposure is related to a particular environment and its chemical–physical characteristics.

In the previous paragraph, we underlined the occurrence and abundance of microplastics in the air and soil web and their transport within the food web, from seafood to beverages and fruits. Considering the high microplastic concentrations detected, the above-mentioned exposure routes can represent important issues for human health.

MPs can have potential adverse effects on human health [210], such as inflammation and secondary genotoxicity [57], and their accumulation can induce or enhance an immune response [111]. Inhaled microplastics can translocate into the respiratory epithelium via diffusion, direct cellular penetration, or active cellular uptake, as reported for other non-biological micro-and nanoparticles [111]. The preliminary effects of microplastic inhalation were studied in workers involved in plastic processing. Histopathological analysis of the lungs of these workers showed interstitial fibrosis and granulomatous lesions, postulated to be acrylic, polyester, and nylon dust [213,214]. The comparison between the number of microplastics absorbed by inhalation and that by ingestion (through the food web) was also reported in the literature [215]. It was observed that the amount of inhaled MPs was from 3 to 15 times higher than the ingested ones, so the levels of MP ingestion by humans are minimal compared to exposure [208].

Dermal contact with microplastics is considered a less significant route of exposure, usually associated with exposure to monomers and additives, such as the endocrine disruptors bisphenol A and phthalates, from the daily use of common appliances [212,216]. For instance, dermal uptake was investigated in rainbow trout. There is evidence for the uptake of 1 μm latex spheres from the surrounding water, with particles localizing and persisting in the surface and sub-surface epidermal cells of the skin and in phagocytes underlying the gill surface [217]. In humans, surgical sutures in medicine, i.e., braided polyester and polypropylene, are known to induce low inflammatory reactions and a foreign body reaction with fibrous encapsulation. Moreover, human epithelial cells suffer oxidative stress from exposure to microplastics and nanoplastics as well [218].

Persorption is considered as a possible route of uptake in the gastrointestinal tract, and it describes the mechanical kneading of solid particles (up to 130 μm diameter) through gaps in the single-layer epithelium at the villus tips and into the circulatory system [219,220,221]. Samples of persorption were obtained using PVC particles (5–110 μm) as a model of non-degradable microparticles, following exposure via feeding or rectal administration, in rats, guinea pigs, rabbits, chickens, dogs, and pigs. Persorption has also been reported in human subjects by using starch particles (200 g) that led to granules being observed in urine, bile, cerebrospinal fluid, peritoneal fluid, and breast milk [222]. Peyer’s patches of the ileum (the third portion of the small intestine) are considered the major sites of uptake and translocation of particles in the gastrointestinal tract. The uptake of plastic microspheres (1–2.2 μm) by Peyer’s patches has been reported in mammalian models such as rats, with an estimation of 60% of PS nanoparticle (60 nm) uptake occurring via Peyer’s patches in rats following 5-day oral dosing. The accumulation of MPs in this compartment could interfere with endogenous microparticle uptake and, consequently, immunosensing and surveillance, compromising local immunity [111].

Obviously, a real estimation of the number of microplastics accumulated in the human body is difficult to obtain. Only a few studies have performed human exposure assessments for MPs, considering total intake from different routes [174,188]. In a recent study, a probabilistic model was used to estimate child and adult exposure to MPs and their accumulation during life [223]. The model considered the ingestion of food (fish, salt, mollusks, and crustaceans) and beverages (tap water, bottled water, beer, and milk) and inhalation through the atmosphere as the main routes of exposure to quantify microplastic intake. Moreover, it included intestinal absorption and biliary excretion to assess MP accumulation. The results highlighted a small contribution of microplastics to total chemical intake compared to other more hazardous contaminants with the same exposure routes, e.g., benzo(a)pyrene, di(2-ethylhexyl)phthalate, 3,3′,4,4′,5-pentachlorobiphenyl, and lead.

Notwithstanding the above, human exposure to MPs remains not-negligible, thus warranting investigations on their effects on humans.

### 5.2. Toxicological Studies and Consequences to Human Health

Adverse effects of environmental exposure to MPs have been mostly studied using marine organisms (77%) and freshwater organisms (23%), while research involving terrestrial organisms is still in its beginnings [224,225]. Often, aquatic organisms at the start of the trophic web are considered since the plastic contamination of such species has become the main topic in bioaccumulation and biomagnification dynamics [226]. For example, a recent study suggested that aquatic microplastic pollution could affect the growth and feeding behavior of *Artemia salina*, a planktonic organism used as a primary food source for many farmed species [227]. In particular, brine shrimps easily internalized 10 µm polystyrene microspheres through filtration. In the absence of a food source, this phenomenon also occurred at low concentrations (1 MPs/mL), whereas, in the presence of a food source (*Dunaliella salina*), microplastics were ingested only at higher concentrations (10–100 MPs/mL). MP uptake, in a dose-dependent manner, caused lower ingestion of a nutritional food source, which led to a developmental delay and a reduction in total body length.

**Figure 3:**
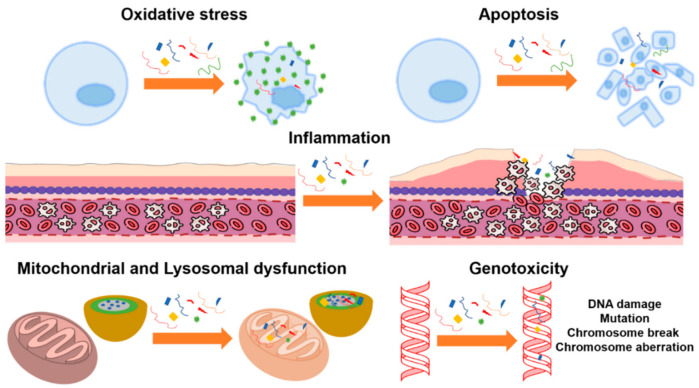
Toxicological effects of polystyrene microparticles on cell cultures: oxidative stress, apoptosis, inflammation, mitochondrial and lysosomal dysfunction, and genotoxicity.

In the last few years, the impact of microplastics and nanoplastics on the human body has been analyzed in both in vitro and in vivo studies [228,229,230,231]. The toxicological hazard of microplastic and nanoplastic exposure to humans through oral ingestion was recently assessed [232]. Several toxicological studies on ingested microplastics are reported in the literature, most of which used polystyrene particles as a benchmark material for more complex microplastics, while only a few examples regarded polyethylene [233,234,235]. However, none of the studies considered human exposure to real-life microplastic samples but evaluated the toxicological effects of PS plastic particles on cell cultures [236,237,238] and also experimental mammal animal models [239,240,241,242]. Moreover, the toxic effects strongly depended on the dose, dose rate, and duration of exposure used in the experiments. The majority of studies on polystyrene particles in cell cultures considered short-term exposure to high microplastic concentrations and showed toxicological effects on parameters such as oxidative stress [218,237,238], inflammation [236,243], mitochondrial dysfunction [238], lysosomal dysfunction [244], and apoptosis [243], whereas only a few studies investigated genotoxicity [245,246,247] (see Figure 3 for the main toxicological effects found in cell cultures).

In contrast to these data, obvious indices of toxicity have not been demonstrated in animal models. Concerning particle size, more toxic results are observed for PS particles that are less than 100 nm with respect to particles larger than 100 nm. Moreover, it was observed that in the functionalization of PS particles, both carboxyl groups (-COOH) and amine groups (-NH_2_) make microparticles more toxic than non-functionalized ones [248]. In the case of polyethylene particles, studies have suggested that these microplastics generate only inflammatory reactions.

Since inhalation is one of the main routes for human exposure to microplastics, some studies have regarded the effects of polystyrene nanoparticles (PS-NPs) on human lung epithelial A549 cells, usually used as a model for human alveolar type II pulmonary epithelium [249,250,251,252,253]. It has been shown that PS-NPs might directly interfere with membrane transporter (P-glycoprotein/MDR1) function in A549 cells, in an amount that depends on their size and surface properties, resulting in a possible influence on the disposition of xenobiotic and endogenous substrates [249]. Moreover, PS-NPs are rapidly internalized by A549 cells, affecting their viability, apoptosis, and cell cycle and disturbing gene transcription and protein expression [250]. The results of this study clearly showed that knowledge of parameters such as the concentration of particles, diameter, and exposure time is necessary to assess the toxicological effects of these particles on human alveolar epithelial A549 cells [250]. Recently, the combined toxicity of PS-NPs and phthalate esters on A549 cells was also investigated. At a greater concentration of PS-NPs, these particles have a dominant role in the combined cytotoxicity observed in mechanisms of oxidative stress and inflammatory reactions [251]. Moreover, to evaluate the potential toxicological effects of microplastics, A549 cells were exposed to polystyrene microplastics. The inhibition of cell proliferation and major changes in cell morphology were observed, confirming that microplastics have a potential for harm to humans [253].

Moreover, A549 cells were used to assess the toxicity of polyvinyl chloride (PVC) microparticles since the inhalation of PVC dust has been associated with pulmonary diseases [254,255]. PVC microparticles produced in vitro cytotoxicity and inflammatory potential for several rat and human pulmonary cells, perhaps due to the presence of residual additives [254,255].

Considering ingestion as another important exposure route for humans, the interaction of PS-NPs with human intestinal cells and intestinal translocation was studied using different in vitro models [256,257]. No significant cytotoxic effect and no adverse effects in the integrity or permeability of the barrier mode were detected [256]. Nevertheless, the studies confirmed the capability of particles to cross the epithelial barrier of the digestive system, which should not be underestimated because of other possible long-term implications [257]. Factors affecting the cellular uptake of PS-NPs were also investigated in different cell lines [258,259,260]. Experiments performed using carboxylated PS-NPs (40 and 200 nm of diameter) revealed that NPs enter cells via active energy-dependent processes for all cell types but exploit different uptake mechanisms depending on cell type [258]. Moreover, surface charge is the main parameter influencing cellular uptake efficiency, whereas compositional elements, aggregation/agglomeration, and protein corona formation results are less relevant [259].

As previously mentioned, MPs can adsorb chemicals such as heavy metals, persistent organic pollutants plasticizers [261], antioxidants and slip agents [262], and some potential pathogens [26,27,28] on their surface, increasing the exposure of humans to toxic chemicals and additives. For example, high concentrations of phthalates and organophosphorus esters have been detected on some beached microplastics based on PP flakes and PS foams [21]. Moreover, MPs containing high levels of potentially bioavailable toxic substances (e.g., lead, cadmium, organochlorine compounds, copper, zinc, and hydrocarbons) may represent a significant ecotoxicological risk for the early life stages of aquatic organisms [263]. As a catalytic surface, microplastics can also affect biochemical processes in organisms, increasing intakes and accumulations of pollutants in organisms [239]. It has been documented how aged plastics strengthen their ability to absorb chemicals because of weathering. Some studies have shown the bioaccumulation of chemicals from plastics in organisms [58] and the presence of potentially pathogenic bacteria on microplastics [27]. Furthermore, biofilm communities on microplastics from three marine ecosystems (the Baltic, Sargasso, and Mediterranean seas) were characterized using high-throughput 16S rRNA gene sequencing. As a result of this investigation, MPs were found to be a possible reservoir of rare and understudied microbes, hence encouraging future studies in this field [28]. MPs colonized by potential pathogens showed a close relationship with coral diseases, with the possibility of disease 4–89% greater than a plastic-free condition [264]. Increased exposure to microplastics can cause immune disorders, neurodegenerative diseases, and cancer [212,265].

Another important issue arises from the ability of microplastics to accumulate over time and to be bio-persistent [111]. An artificial in vitro digestion protocol (considering the three digestive compartments: mouth, stomach, and intestine) allowed us to analyze the effects of gastrointestinal passage on the physiochemical particle characteristics of the most environmentally abundant materials, such as PE, PP; PVC, PET, and PS [266]. The SEM results demonstrated a high resistance of all MPs to the artificial digestive juices compared to the influence of the positive controls of hydrochloric acid, nitric acid, and acetone. Moreover, a cross-disciplinary review discusses and evaluates the potential impacts of microplastics on human health through diet and environment [111]. There was no evidence of increased cancer risk for nylon flock workers, although they had a higher prevalence of respiratory diseases (dyspnea, coughing, reduced lung capacity, and wheezing) [267,268,269].

Ultimately, several toxic effects of plastic particles were observed, but they mainly occurred for particle sizes smaller than 5 µm at a concentration much higher than human exposure, demonstrating hazard rather than risk for human health, so the same effects at low concentration must be verified. Epidemiological studies must be carried out to assess the real consequence of microplastic contamination at concentrations in the range of human exposure.

### 5.3. Future Trends: Occurrence in Body Fluids and Related Effects

The above-discussed papers assert an elevated microplastics intake, so a deeper investigation on the translocation and accumulation of MPs in the human body is needed to better characterize their potential to harm humans. In fact, MP occurrence and detection in the fluids of the human body remain a poorly investigated field but could be very useful for assessing the interaction of plastic particles with the human body.

In a recent study, the presence of microplastics in human stool was investigated [270]. All samples analyzed (from 3 men and 5 women) contained microplastics (size range of 50–500 μm), with polypropylene and polyethylene terephthalate as the most abundant polymers and an average concentration of 2 particles/g of fecal matter.

Surprisingly, microplastic fragments were also detected in human placenta samples collected from six consenting women with uneventful pregnancies [271]. Analysis by Raman microspectroscopy was able to detect 12 pigmented microplastic fragments (5–10 μm in size) in 4 placentas, mainly polypropylene. The presence of microplastics in the human placenta is a matter of great concern, so further studies should be performed to evaluate if it can result in harmful effects on pregnancy.

These results encourage the search for microplastics in human fluids, and other investigations should be carried out to assess how microplastics interact with the human body and reach its fluids. A significant issue to consider in microplastic determination is the contamination of samples due to airborne microplastics. Consequently, great attention must be paid to sample treatment to avoid the misidentification of microplastics in human samples, especially for low size particles (<10 mm), and enlarge the number of samples collected. Therefore, in the near future, new methodologies should be developed to count and characterize MPs in body fluids, avoiding interferences as much as possible.

Moreover, more international and cross-disciplinary research focusing on the toxicology of these particles is urgently needed to fully understand the long-term effects on humans and help health organizations to provide prevention guidelines.

## 6. Conclusions

Plastic microparticle accumulation in the environment leads to stress on ecosystems. In this review, we analyzed the most recent literature related to microplastics in the environment and food, the potential route of exposure for humans, and toxicological effects. We have underlined how several literature studies have detected high microplastic concentrations in the environment, with the consequent transport of these particles within the food web, from seafood to beverages and fruits. A higher number of studies have researched the contamination of sea products, drinking water (raw, treated, and stored in different packages), salts for human consumption, and honey, sugar, fruit, and chickens. All these studies suggest a not-negligible ingestion of microplastic particles through food consumption. Several toxic effects of ingested microplastics, mainly PS, were reported to occur at high microplastic concentrations compared to human exposure, demonstrating a hazard for human health. Further studies must be performed to assess the real consequences of microplastic contamination at concentrations in the range of human exposure. Increasing awareness of the potential and growing risks to human health has not been accompanied by considerable efforts to establish the influence of microplastic abundance on human health in vivo, most probably because of the lack of standardization of sampling methodologies and the separation of MPs. This review aims to guide future researchers into a deeper investigation of the processes involved in MP uptake, the potential mechanisms of toxicity, and health effects.

## Figures and Tables

**Table 1:** Occurrence and quantification of microplastics in foods and beverages.

Sample	Sampling Location	Abundance Average (Range)	Size Range	Polymer Type	Ref.
Seafood					
European anchovies	Mediterranean Sea (Gulf of Lions)	-	0.124–0.438 mm	PE, styrene/acrylonitrile	[123]
Bivalves (*Mytilus edulis* and *Crassostrea gigas*)	Germany and Brittany (FR)	0.36–0.47 particles g^−1^	>0.005 mm	-	[136]
Mussels (*Mytilus edulis*)	French–Belgian–Dutch coastline (FR, BE, NL)	0.2–0.5 particles g^−1^	0.015–1 mm	-	[135]
Dogfish, hake, red mullet	Galician coast, Cantabrian coast, Gulf of Cadiz, Spanish Mediterranean coast (ES)	1.56 ± 0.5 particles/individual	0.38–3.1 mm	-	[121]
Semipelagic fish	Mallorca and Eivissa (Balearic Islands, ES)	3.75 (2.47–4.89) particles/individual	0.5 mm	-	[132]
Pelagic and demersal fish	Plymouth (UK)	1.90 particles/individual	0.13–14.3 mm	PA, cellulose, RY	[131]
Benthic and pelagic fish	Portugal coast (PT)	0.27 ±0.63 particles/individual	0.217–4.81 (average 2.11) mm	PP, PE, ALK, RY, PES, NY	[133]
Different fish species	Mediterranean coast of Turkey (TR)	2.36 particles/individual	average 0.656 mm	polystyrene: isoprene, PE, PP	[126]
Red mullet (*Mullus surmuletus*)	Palma, Port d’Andratx, Port d’Alcúdia, Cala Ratjada and Santanyí (Mallorca, ES)	(0.32–0.68) particles/individual	-	PET, CPH, Polyacrylate, PAN	[120]
Deep benthic invertebrates	Rockall Trough, Scotland (UK)	1.582 ± 0.448 particles g^−1^	0.023–6.25 (average 1.191) mm	ALK, PES	[124]
Benthic organisms	South Yellow Sea (North China and South Korea, CN, KR)	(1.7–47.0) particles g^−1^	0.05–5 mm	PP, PE, PS, PET, NY	[137]
Mussels (*Mytilus edulis*)	Coastal water of China (CN)	0.9–4.6 particles/individual 1.5–7.6 particles g^−1^	0.033–4.7 mm	CPH, PET, PES	[129]
Different fish species	Rapa Nui (Easter Island, CL)	2.5 particles/individual	0.2–5 mm	PE, PP	[134]
Bivalve (oyster, mussel, Manila clam and scallop)	South Korea (KR)	0.97 (0–2.8) particles/individual 0.15 (0–1.8) particles g^−1^	0.1–0.2 mm	PE, PP, PS, PES, PEVA, PET, PUR	[122]
Deep-sea fish	South China sea (CN)	Stomach: 1.96 particles/individual and 1.56 particles g^−1^; Intestine: 1.77 particles/individual and 4.89 particles g^−1^	<1 mm	CPH, PA, PET,	[138]
Indian white shrimps	Kochi, Southwest India (IN)	0.39 particles/individual 0.04 particles g^−1^	0.157–2.785 mm	PA, PES, PE, PP	[125]
**Salt**					
Sea salt, lake salt, rock/well salt	China (CN)	550–681 particles kg^−1^ (sea salt) 43–364 particles kg^−1^ (lake salt) 7–204 particles kg^−1^ (rock/well salt)	0.1–1 mm	PET, PE, PB, PP, PES, CPH	[170]
Ocean salt, sea salt, rock salt	United States (USA)	47–806 particles kg^−1^	0.1–5 mm		[166]
Sea salt	Italy (IT)	1600–8200 particles kg^−1^	0.004–2.1 mm		[168]
Sea salt	Croatia	13,500–19,800 particles kg^−1^	0.015–4.6 mm	PP	[168]
Sea and lake salt	Australia (AU), France (FR), Iran (IR), Japan (JP), Malaysia (MY), New Zealand (NZ), Portugal (PL), South Africa (ZA)	1000–10,000 particles kg^−1^	0.2–1 mm	PE, PET (AU) PP, PET (FR) PP (IR) PE, PET (JP) PP (MY) PE (NZ) PET, PP (PL) PET (ZA)	[164]
Sea salt	Indonesia (ID)	100 particles kg^−1^	0.1–2 mm	PE, PET, PP	[165]
**Drinking water, soft drinks, and milk**					
Drinking water	Oldenburg-East-Frisian water board, Germany (DE)	0–7000 particles L^−1^	0.05–0.150 mm	PES, PVC, PE, PA, EP	[181]
Drinking water	Germany (DE)	11 ± 8 particles L^−1^ (beverage cartons) 118 ± 88 particles L^−1^ (returnable plastic bottles)	0.005–0.1 mm	PET, PE, PA, PP	[184]
Drinking water	Bavaria, Germany (DE)	4889 ± 5432 particles L^−1^ (reusable PET bottles) 2649 ±2857 particles L^−1^ (single-use PET bottles) 3074 ±2531 particles L^−1^ (glass bottles)	0.001–0.01 mm	Styrene-butadiene copolymer, PP, PE, PET	[182]
Drinking water	Czech Republic (CZ)	338 ± 76 to 628 ± 28 particles L^−1^ (treated water) 1473 ± 34 to 3605 ± 497 particles L^−1^	0.001–0.1 mm	PBA, PE, PET, PMMA, PP, PS, PTT, PVC (raw water) PAAm, PE, PET, PP, PVC (treated water)	[183]
Cold tea, soft drinks, energy drinks, beers	Mexico City, Mexico (MX)	11 ± 5.26 particles (cold tea) 40 ± 24.53 particles (soft drinks) 14 ± 5.79 particles (energy drinks) 152 ± 50.97 particles (0–28 ± 5.29 particles L^−1^) (beers)	0.1–3 mm	PA, PET, PEA, ABS	[189]
Beer	Germany (DE)	2–79 particles L^−1^ (fibers) 12–109 particles L^−1^ (fragments) 2–66 particles L^−1^ (granules)			[192]
Beer	Germany (DE)	16 ± 15 particles L^−1^ (fibers) 21 ± 16 particles L^−1^ (fragments) 27 ± 10 particles L^−1^ (granules)			[191]
Beer	USA	0–14.3 particles L^−1^	0.1–5 mm		[166]
Milk	Mexico City, Mexico (MX)	6500 particles m^−3^	0.1–5 mm	Polysulfone	[195]
**Honey, sugar, and fruit**					
Honey	Germany (DE), France (FR), Italy (IT), Spain (ES)	166 ± 147 particles kg^−1^ (fibers) 9 ± 9 particles kg^−1^ (fragments)	0.01–9 mm		[197]
Honey	Germany (DE)	10–336 particles kg^−1^ (fibers) 2–82 particles kg^−1^ (fragments)	0.01–several mm		[209]
Honey	Switzerland (CH)	32–108 particles kg^−1^ (fibers) 8–28 particles kg^−1^ (other)		PET	[198]
Honey	Ecuador (EC)	54 particles L^−1^ (industrial honey) 67 particles L^−1^ (craft honey)	0.013–0.25 mm	PP, PE, PAAm	[199]
Sugar	Germany (DE), France (FR), Italy (IT), Spain (ES)	217 ± 123 particles kg^−1^ (fibres) 32 ± 7 particles kg^−1^ (fragments)			[197]
Fruit and vegetables	Catania, Italy (IT)	52,600–307,750 particles g^−1^ (apples) 98,325–302,250 particles g^−1^ (pears) 65,025–201,750 particles g^−1^ (cabbages) 26,375–75,425 particles g^−1^ (lettuce) 72,175–130,500 particles g^−1^ (carrots)	0.00156–0.00319 mm (apples) 0.00187–0.00259 mm (pears) 0.00186–0.00295 mm (cabbages) 0.00218–0.00278 mm (lettuce) 0.00136–0.002 mm (carrots)		[200]
**Chicken, cows, and pigs**					
Chicken feces	Campeche, SE Mexico (MX)	129.8 ± 82.3 particles g^−1^	0.1–5 mm		[201]
Chicken gizzards	Campeche, SE Mexico (MX)	10.2 ± 13.8 particles g^−1^	0.1–5 mm		[201]
Poultry, pigs, cows	South China (CN)	902 ± 1290 particles kg^−1^ (pig manure) 667 ± 990 particles kg^−1^ (poultry manure) 74 ± 129 particles kg^−1^ (cow manure) 139 ± 115 particles kg^−1^ (pig feeds) 96 ± 109 particles kg^−1^ (poultry feeds) 36 ± 63 particles kg^−1^ (cow feeds)	<5 mm	PP, PE, PET	[204]

Abbreviations. MO: microscope; FTIR: Fourier transform infrared spectroscopy; RMS: Raman spectroscopy; SEM: scanning electron microscope; FTIR-ATR: Fourier transform infrared spectroscopy–attenuated total reflectance; SEM-EDX: scanning electron microscopy with energy dispersive X-ray analysis; PE: polyethylene; PP: polypropylene; PS: polystyrene; PVA: polyvinyl alcohol; PVC: polyvinyl chloride; PES: polyester; PET: polyethylene terephthalate; PUR: polyurethane; PA: polyamide; NY: nylon; CPH: cellophane; RY: rayon; EP: epoxy resin; ALK: alkyd resin; PAN: polyacrylonitrile; PMMA: poly(methyl methacrylate); ABS: acrylonitrile butadiene styrene; PEVA: polyethylene-vinyl acetate; PAAm: polyacrylamide; PB: poly(1-butene); PBA: polybutylacrylate; PTT: polytrimethylene terephthalate; PEA: poly(ester-amide).

**Table 2:** Estimated intake of microplastics through inhalation, food and beverages, and packaging.

Sample	Origin	Estimated Intake	Ref.
Air (inhalation)	Europe (UE)	26–130 particles/day/capita 272 particles/day/capita	[210,211]
Dust	Tehran, Iran (IR)	107–736 particles/year/capita (adults, normal exposure) 353–2429 particles/year/capita (adults, acute exposure) 644 particles/year/capita (children, normal exposure) 3223 particles/year/capita (children, acute exposure)	[34]
Seafood	Europe and American countries	518–3078 particles/year/capita	[141]
Seafood	UK Other countries such as France, Belgium, and Spain	123 particles/year/capita (UK) 4620 particles/year/capita	[208]
Salt	Australia, France, Iran, Japan, Malaysia, New Zealand, Portugal South Africa	37 particles/year/capita	[164]
Salt	Turkey	249–302 particles/year/capita (sea salt) 203–247 particles/year/capita (lake salt) 64–78 particles/year/capita (rock salt)	[162]
Salt	North Sea Salt, Celtic Sea Salt, Mediterranean Sea Salt Mediterranean Sea Salt Utah Sea Salt Himalayan Rock Salt Mined Hawaiian Sea Salt Ocean Baja Sea Salt Ocean Atlantic Sea Salt Ocean Pacific Sea Salt	40–680 particles/year/capita	[166]
Salt	Spain	510 particles/year/capita	[163]
Salt	China (CN)	1000 particles/year/capita	[170]
Drinking water	Asia, USA, and Europe	3000–4000 particles/year/capita	[187]
Drinking water	America	4000 particles/year/capita (consumers of tap water) 90,000 particles/year/capita (consumers of water from plastic bottles)	[188]
Drinking water, salt, and beer	USA	5800 particles/year/capita	[166]
Milk (Infant exposure)	Asia, Europe, America, Oceania, Africa	527,000 and 893,000 particles/day/capita (Asia and Africa) 2,100,000 particles/day/capita (Oceania) 2,280,000 particles/day/capita (North America) 2,610,000 particles/day/capita (Europe)	[196]
Fruit and vegetables	Italy (IT)	29,600–1,416,000 particles/kg/day	[200]
Meat (food packaging)	France (FR)	0.1–515.2 mg/year/capita	[205]
Take-out containers	China (CN)	2977 particles/year/capita	[207]

## Data Availability

No new data were created or analyzed in this study. Data sharing is not applicable to this article.
